# Multiple Venous Thromboses and Renal Failure in Behcet's Disease: A Case Report and Review of the Literature

**DOI:** 10.7759/cureus.57560

**Published:** 2024-04-03

**Authors:** Sara Lalouly, Oumaima EL kaoua, Mariam Chettati, Wafaa Fadili, Inass Laouad

**Affiliations:** 1 Department of Nephrology, Centre Hospitalier Universitaire (CHU) Mohammed VI, Arrazi Hospital, Marrakech, MAR; 2 Faculty of Medicine and Pharmacy of Marrakech, Cadi Ayyad University, Marrakech, MAR

**Keywords:** fatal, internal medicine. clinical nephrology, thrombo-embolic disease, end-stage renal failure, behcet

## Abstract

Behcet's disease (BD) is a systemic condition of unknown etiology, characterized by a wide clinical polymorphism. Vascular involvement in BD is rare and can be revealing in many cases. We present an advanced case of BD with multiple venous thromboses associated with urgent dialysis-dependent end-stage chronic renal failure. This case highlights the complexity of managing BD, emphasizing the challenges associated with multiple thromboses and the crucial importance of early diagnosis to optimize the management of this systemic disease.

## Introduction

Behcet's disease (BD) is a systemic condition of unknown etiology, characterized by significant clinical polymorphism with a particular frequency of dermatological manifestations representing three out of the four criteria in the disease classification adopted by the International Study Group (ISG) [1]. Vascular involvement in BD is severe and can affect vessels of any size and location, including both arteries and veins [2]. Multiple venous thromboses and renal involvement are relatively rare manifestations of BD. We present an advanced case of BD with multiple venous thromboses associated with urgent dialysis-dependent end-stage chronic renal failure.

## Case presentation

Observation

**Figure 1:**
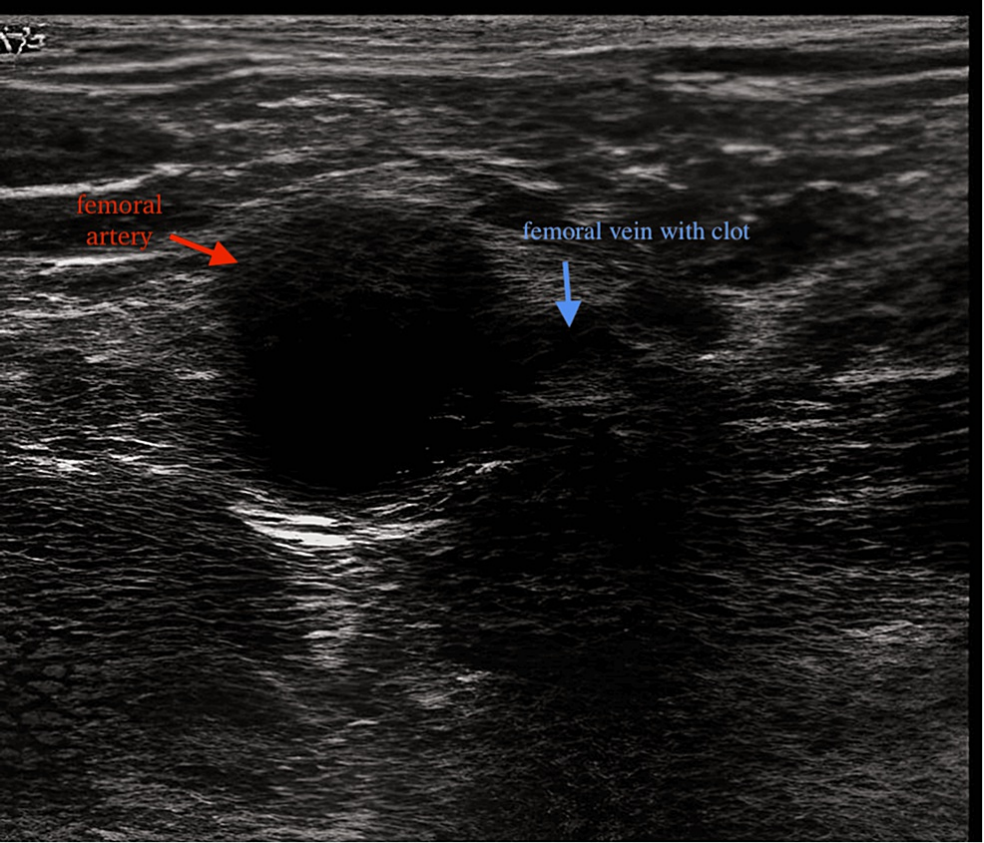
Thrombosis of the right femoral vein on Doppler ultrasound.

A 46-year-old patient from southern Morocco, who had been treated for varicose veins of the lower limbs for 14 years with acetylsalicylic acid 600 mg daily and hesperidin 1 g daily, was diagnosed with end-stage chronic renal failure a month ago. The patient presented with clinical and biological uremic syndrome and was referred to the nephrology department after the failure of placing a hemodialysis catheter. Doppler ultrasound revealed superficial and deep venous thromboses in the upper and lower limbs, as well as bilateral jugular and subclavian veins (Figure 1).

The medical history revealed recurrent oral aphthosis. Upon clinical examination, the patient was found to be conscious and hypertensive at 160/90 mmHg, with initially preserved diuresis. Bilateral soft edema of the lower limbs extending to the ankles was observed, along with ocher dermatitis on the anterior surface of both legs, and bipolar aphthosis. The laboratory tests revealed an impure nephrotic syndrome with 24-hour proteinuria of 6 g/g and hypoalbuminemia of 23 g/L associated with severe renal insufficiency with a calculated glomerular filtration rate (GFR) of 1.8 mL/minute (Modification of Diet in Renal Disease [MDRD] formula). The rest of the investigation showed normochromic normocytic anemia at 7.4 g/dL, hypocalcemia at 58 mg/L, hyperphosphatemia at 117 mg/L, as well as an inflammatory syndrome (C-reactive protein [CRP] 56 mg/L) and aseptic leukocyturia in urine analysis.

Regarding the radiological assessment, a renal ultrasound was performed, revealing kidneys consistent with chronic nephropathy.

Diagnosis

Given the presence of oral and bipolar aphthous ulcers and vascular involvement, we suspected BD associated with terminal renal failure. A pathergy test was performed that came back positive, and an ophthalmologic examination ruled out the presence of anterior uveitis, a common ocular manifestation of BD.

Treatment and evolution

The patient was placed on corticosteroid therapy at a dose of 1 mg/kg/day, along with azathioprine 50 mg/day and colchicine 1 mg/day. The course was marked with a decrease in CRP levels within eight days, but a significant rise in muscle enzymes, exceeding 10 times the normal levels for creatine phosphokinase (CPK). Peritoneal dialysis (PD) was initiated seven days after the placement of a PD catheter under local anesthesia. However, it was complicated after 15 days of daily sessions by leakage and superinfection of the PD fluid with multidrug-resistant *Acinetobacter baumannii*. Despite appropriate antibiotic therapy, the patient succumbed to septic shock.

## Discussion

This is an unusual case report of a patient with BD with multiple venous thromboses complicated by acute renal failure and requiring dialysis. Vascular involvement is a serious complication of BD and is the leading cause of death in patients with the disease [3]. The incidence of vascular involvement in BD ranges from 7% to 29%, with venous thrombosis being the most common manifestation. In a study of 1200 patients with BD, 12.8% had venous thrombosis but only 0.8% had jugular vein thrombosis, making it an unusual manifestation of the disease [4]. Pathergy test positivity and eye involvement are commonly seen in patients with vascular involvement [3]. But in this case, there was no history of eye involvement.

Renal failure is a rare complication of BD. According to a study in Turkey, the prevalence of BD among dialysis patients is very low, at only 0.07% among 20,596 patients. This low prevalence may be because many people with BD die within a few weeks or months after being diagnosed with end-stage renal disease. Renal amyloidosis is the most common cause of chronic renal failure in these patients. However, it is also possible that long-term use of acetylsalicylic acid may lead to interstitial nephritis. Additionally, renal vein thrombosis should also be suspected in patients with vascular involvement [5]. There are numerous issues associated with arteriovenous fistulas or temporary vascular access according to literature. To prevent complications related to temporary vascular access, it is essential to diagnose renal disease early and properly manage kidney failure [5-6]. There are only a few reported cases of using peritoneal dialysis in patients with BD, and these cases have indicated a high risk of septic thrombosis of the catheter [7]. In our case, peritoneal dialysis was complicated by leakage and multi-resistant germ peritonitis that ultimately led to the patient's death.

Aggressive surgical or interventional treatment is not effective in altering the course of the pathology itself. So medical treatment is necessary to suppress the exacerbations. However, managing the disease can be difficult, especially when there are multiple sites of involvement. Immunosuppressive treatment is often the best option, and colchicine and thalidomide can be effective for mucocutaneous symptoms. Combination therapy with corticosteroids and nonselective immunosuppressive drugs (azathioprine, methotrexate, chlorambucil, cyclosporine A, tacrolimus, or cyclophosphamide) may be necessary when vital organs are involved [8]. There is some evidence that interferon-alpha and anti-tumor necrosis factor (anti-TNF) therapy can be effective, but more research is needed to determine the best treatment options for BD complicated by concurrent thromboses [9-10]. New treatment modalities should be explored and implemented when necessary.

## Conclusions

BD is a systemic condition with a significant clinical polymorphism that can complicate end-stage chronic renal failure. Managing this condition is challenging due to multiple thromboses, making the creation of vascular access difficult. Hence, the importance of early diagnosis and close monitoring cannot be overstated.
